# Hostility, compassion and role reversal in West Virginia’s long opioid overdose emergency

**DOI:** 10.1186/s12954-020-00416-w

**Published:** 2020-10-12

**Authors:** Jeff Ondocsin, Sarah G. Mars, Mary Howe, Daniel Ciccarone

**Affiliations:** 1 Heroin in Transition Study, UCSF Department of Family and Community Medicine, University of California, 500 Parnassus Avenue, Milberry Union East, 3rd Floor, San Francisco, CA 94143 USA; 2 Homeless Youth Alliance, PO Box 170427, San Francisco, CA 94117 USA

**Keywords:** Injection drug use, Overdose, Opioids, Harm reduction, Hostility, Compassion, Risk environment

## Abstract

**Background:**

West Virginia is a largely rural state with strong ties of kinship, mutual systems of support and charitable giving. At the same time, wealth inequalities are extreme and the state’s drug overdose fatality rate stands above all others in the USA at 51.5/100,000 in 2018, largely opioid-related. In recent years, harm reduction services have been active in the state but in 2018 Charleston’s needle and syringe program was forced to close. This paper considers the risk environment in which the state’s drug-related loss of life, and those attempting to prevent it, exist.

**Methods:**

This rapid ethnographic study involved semi-structured interviews (n = 21), observation and video recordings of injection sequences (n = 5), initially recruiting people who inject heroin/fentanyl (PWIH) at the Charleston needle and syringe program. Snowball sampling led the research team to surrounding towns in southern West Virginia. Telephone interviews (n = 2) with individuals involved in service provision were also carried out.

**Results:**

PWIH in southern West Virginia described an often unsupportive, at times hostile risk environment that may increase the risk of overdose fatalities. Negative experiences, including from some emergency responders, and fears of punitive legal consequences from calling these services may deter PWIH from seeking essential help. Compassion fatigue and burnout may play a part in this, along with resentment regarding high demands placed by the overdose crisis on impoverished state resources. We also found low levels of knowledge about safe injection practices among PWIH.

**Conclusions:**

Hostility faced by PWIH may increase their risk of overdose fatalities, injection-related injury and the risk of HIV and hepatitis C transmission by deterring help-seeking and limiting the range of harm reduction services provided locally. Greater provision of overdose prevention education and naloxone for peer distribution could help PWIH to reverse overdoses while alleviating the burden on emergency services. Although essential for reducing mortality, measures that address drug use alone are not enough to safeguard longer-term public health. The new wave of psychostimulant-related deaths underline the urgency of addressing the deeper causes that feed high-risk patterns of drug use beyond drugs and drug use.

## Introduction

Amidst the greatest loss of life from drug overdoses in the recorded history of the USA, the death rate of the state of West Virginia stands above all others. West Virginia (WV) has experienced statistically significant increases in drug overdose deaths every year since 2013 with an age-adjusted death rate of 57.8 per 100,000 in 2017 when this study was conducted [1]. These increases have occurred across multiple drug categories—including heroin, synthetic and prescription opioids and psychostimulants [2, 3]—with significant increases in overdose deaths attributed to multiple drugs [3]. Synthetic opioids, including fentanyl and its analogs, have been attributed to an increasing number of deaths in the state, with rates almost tripling between 2015 and 2017 (12.7 to 37.4 per 100,000) [1, 2]. Prescription opioid deaths remain high (19.7 per 100,000 in 2016) [2] with a slight decline in 2017 (17.2 per 100,000) [1], the highest in the USA for both years.

Other than Berkeley County in West Virginia’s eastern panhandle, overdose deaths are concentrated in the southern portion of the state and along the Ohio border, including the counties of Kanawha (home to Charleston, the state capitol) and Cabell (county seat of Huntington) [3] where the research for this paper was carried out. Cabell County had an opioid overdose death rate of 72.7 per 100,000 in 2017, almost five times the national average [1], eclipsing Kanawha County’s rate of 30.6 per 100,000 [4].

A rural state situated in the US Appalachian region, the population of West Virginia is approximately 93% Caucasian and the poverty rate 17.9% [5]. Poverty status in the USA is calculated by a comparison of annual income to poverty thresholds determined by family size, number of children and age of the householder [6]. While this mean poverty level is higher than the national average, there are also wide inequalities of income within its lines—in 2016 the city of Charleston had a 20% poverty rate, while Huntington’s was 30.8%, with median household income in both cities below the national average [5]. Huntington has particularly low median household income (half the national average) and higher rates of disability than both the West Virginia and national average [5]. Patterns of land ownership have contributed to these disparities. Historically, much of the state’s land and natural resources have been owned and controlled by absentee owners, often coal monopolies who have acquired not only land but also socio-political power while diminishing community power [7, 8]. This trend has continued into the current century along with the expansion of timber interests [9, 10]. This power imbalance and lack of local accountability has resulted in some of the most lax environmental and labor regulations in the USA [11]. Many industrial accidents have occurred in recent years in the chemical and coal industries, causing deaths, injuries and polluted ground water [10].

In spite of high poverty rates, ties of kinship and mutual systems of support are particularly strong [12]. West Virginia ranks among the top states in time spent talking with or performing favors for neighbors and family [13]. In 2016, the Charleston-Huntington metro area ranked among the top metros nationwide in the percentage of those donating money to charities and nonprofit organizations, including churches and religious organizations [14].

There has been considerable focus on the high levels of opioid prescribing that preceded the current heroin and fentanyl epidemic. Earlier research has documented the increased harm resulting to opioid dependent patients who resorted to the illicit street opioid market after their opioid pill prescriptions were discontinued [15]. However, while opioid prescribing rates have fallen nationwide, West Virginia has continued to see higher than average rates of opioid prescriptions. In 2016, US opioid prescribing rates stood at 66.5 prescriptions per 100 people, but rates in Kanawha and Cabell counties were nearly twice the national average (111.2 and 122.3, respectively) [16]. *OxyContin* use in Appalachia has been independently associated with injection initiation and rapid injection transition [17] while prescription opioid injection has been associated with non-fatal overdose and HCV infection [18, 19]. A number of demand factors have been proposed for the high levels of prescription opioid use in West Virginia, including out-migration of young adults more resilient to problem drug use and economic stressors [20].

New guidelines governing naloxone distribution were issued by the West Virginia state legislature in 2015, which maintained naloxone as a prescription medication and mandated stringent reporting requirements not placed on other controlled substances [21]. A latent class analysis using data from 2018 found that polysubstance use among people who inject drugs in Cabell County was highly prevalent and was associated with both overdose and receiving take-home naloxone [22]. Needle and syringe programs (NSP) have expanded in Appalachia since 2013, with nine open in West Virginia in August of 2017, although access is limited in many counties [23]. At the time of our study, Huntington and Charleston both had functional NSPs to serve their drug using populations. Since then, West Virginia has experienced several HIV clusters, with injection-related HIV cases reported in Wheeling, WV in 2018 [24] and again in Cabell County and surrounding counties, with 85 incident cases in Cabell County as of April 2020 [25, 26].

Statistical data on rates, locations and trends in overdose deaths are essential for targeting public health responses but do not illuminate the human processes by which risk is produced and experienced. The socially situated nature of risk requires ground-up or ‘emic’ perspectives to elucidate “how risk environments are *experienced* and *embodied* as part of everyday practices” [27]. While individual behavioral adaptations to increased overdose risk have been observed, including monitoring drug appearance, texture and color, relying on a trusted dealer or using a variety of drug sampling techniques [28–32], risk is unequally distributed across populations. Risk environment approaches take into account these socio-structural and geographic determinants of health and risk-taking [31, 33–36]. A risk environment approach “encourages us to think about the social situations and places in which harm is produced and reproduced” [35], allowing for the redistribution of responsibility for harm between individuals and macro-structural socio-economic forces [27].

West Virginia leads the nation in overdose death rate and both counties where participants were interviewed for this study were considered to be at risk for an HIV outbreak among people who inject drugs [37]. Statistical data tell only part of the overdose story. This study asked the question ‘What on-the-ground environmental factors among PWIH could be contributing to this overdose rate?

To this end, we will present qualitative findings from semi-structured interviews, ethnographic observation and filmed injection sequences with a focus on the perspectives of people injecting heroin and drugs sold as heroin, including fentanyl, (‘heroin’ hereafter) in West Virginia. We conceptualize harm as emanating from the ‘structural risk environment’. This is a framework that views individual behavior within wider social, political and environmental contexts in which harms result from interactions between micro-, meso- and macro-level forces [27, 35].

## Methods

The “Heroin in Transition” (HIT) study engages in “hotspot” research where teams of ethnographers travel to US locations upon reports of unusual/novel heroin or high rates of overdose. HIT uses rapid ethnography to gain insight into larger changes and developments occurring in the US heroin supply as they manifest at the local level. Ethnography has been widely utilized in research among people who use drugs [38], and rapid ethnography builds upon this method while using a highly focused approach trying to capture an emic perspective on heroin use across the USA [39]. These data are compared with the observations of the ethnographic team based on their experience of heroin use in other locations, as described elsewhere in studies using rapid ethnography to study the evolving US opioid epidemic [28–30].

### Recruitment and data collection

After contact from NSP administrators and upon receiving reports of unusually high rates of overdose, PI (DC) and ethnographer (JO) were invited to attend the February 2017 meeting of the West Virginia Association of Health Departments and conduct exploratory fieldwork at the Charleston NSP. This initial trip was conducted to determine the feasibility of future fieldwork in the state and how best to engage local PWIH.

In September 2017, DC and ethnographers (JO and MH) returned to Charleston, WV to interview local PWIH. Recruitment was conducted at the Charleston NSP, and contacts were made to conduct interviews in the following days. Some snowball sampling occurred, which led the team to Huntington, WV to interview acquaintances of a Charleston participant. Some respondents were also interviewed in small towns located in Kanawha County. The University of California, San Francisco Institutional Review Board, approved the study protocol. A US Federal Certificate of Confidentiality issued by the National Institutes of Health/National Institute on Drug Abuse protects the data and its collection.

Study eligibility required participants to be at least 18 years of age and self-reported current injectors primarily using heroin. Exclusion criteria covered individuals who were intoxicated or otherwise unable to give informed consent or answer questions reliably. Informed consent was given by all study participants. Data were collected through semi-structured interviews, ethnographic observation documented in fieldnotes and visual recording (photographic & video) of heroin preparation and consumption. These three methods have been described in detail elsewhere [27–29, 37]. Video and photographic recording allows the researchers to view the entirety of the injection process, evaluate health risks and identify levels of injection technique and skill without solely relying on self-report about injection ability. Video captured details about drug appearance and preparation, equipment commonly available, as well as venous health, injection site and level of injection skill, in addition to observation of several instances of partner injection for those who required assistance to inject. Wherever possible, the participants’ usual living environment was used as the setting. The use of video recordings in drug-related research is unusual but has the valuable advantage of allowing the simultaneous capture of many details that may be relevant to drug-related risks and can be reviewed by the ethnographers after the research visit.

### Sample characteristics

This was a non-random convenience sample. Across all locations, 19 current PWIH were interviewed of whom 9 were male and 10 were female. 18 participants were Caucasian and 1 was African-American. Participants have been assigned pseudonyms for confidentiality.

### Analysis

Corrected transcripts were read and material contextualizing the West Virginia risk environment was extracted based on initial categories developed from the semi-structured interview guide — drug use history, progression to heroin injection, experience using heroin/fentanyl, drug discernment, polysubstance use and overdose experience. Analysis used an inductive method, with patterns discerned from interviews, fieldnotes and video recordings in order to make inferences about the experience of PWIH in West Virginia. JO, SM and DC discussed this material and developed these thematic categories for further analysis. Final thematic categories that were included in this paper consisted of heroin initiation, experience of overdose, interactions with law enforcement and emergency responders, perspectives about the NSP, experience injecting and social/kinship relations. These categories contributed to the analysis of injection skill and ability. MH analyzed drug preparation and injection techniques captured in video recordings to review micro-health risks at the point of use and examine injection technique. These findings were then compared with the self-report data to confirm or contradict participant accounts of their injection skill and knowledge. Analysis prioritized the participants’ experience using drugs sold as heroin but ethnographic observations from fieldnotes were included to contextualize participant data.

Following the study visit, the Charleston NSP was often in the local news cycle due to its politicization by then-mayor Danny Jones and his preferred successor candidate ahead of the city’s mayoral election in 2018. The authors continued to monitor operational changes implemented at the NSP through local and national news coverage as well as through personal communications with the director of the NSP. PI Ciccarone was directly involved in some of these developments, contributing to a review of the state Health Department’s findings [40]. Following the closure of the NSP, telephone interviews were carried out with John C Linton, Associate Vice President and Dean of the West Virginia University School of Medicine-Charleston, in light of his expertise in emergency responses in the area and the former director of the Charleston NSP, Dr. Michael Brumage.

## Results

### The long shadow of prescription opioids

All our respondents had extensive experience using prescription opioid pills and most (n = 16) initiated opioid use with them while only three initiated with heroin. While transitions from opioid pills to heroin were common earlier in the US opioid epidemic [15], after 2010 new users in other US states were more commonly initiating opioid use with heroin [41]. However, in the sample interviewed here, this trend was less evident, with prescription opioids remaining an important route to heroin. Interview data suggested that, while prescription opioids remained more available than in other locations, heroin had quickly captured a large share of the opioid-using market.

Nicole from Charleston, in her thirties and injecting for six months, thought that heroin had achieved primacy among her peers, stating ‘Everybody is on heroin. Heroin, heroin, heroin.’ Christine, in her 30s, and injecting for about five years, concurred that heroin use had become the new norm: “Knowing about and doing heroin is more common than […] smoking weed […] it’s like, weed back in the day is what heroin is nowadays.” The sudden widespread adoption of heroin in southern West Virginia, reported by interviewees only in the previous five years (2012–2017), has brought distinctive problems and devastating consequences.

Scott from South Charleston, in his twenties and injecting for five years, vividly illustrated the human toll of West Virginia’s overdose crisis:

Q: Anything else you think we should know about heroin or related drugs in this part?

A: …I know that it’s killed a lot of people around here. I do know that. The majority of my graduating class, that never even used drugs when I was even in school, that even so much as smoked pot, are dead.[…] I graduated in 2007… probably when I go to my 20th year reunion—if I make it that long, hopefully—there are probably less than half of them.

## The law and emergency services

As alluded to in the title of this paper, some interviewees reported experiencing an extreme lack of compassion to people overdosing on opioids. Tina, in her 30s and injecting for 8 years, said, ‘*A lot of people I’ve heard them say—that don’t know that I do it, you know— “You should not give the Narcan and just let it sort itself out. Just let them all overdose.*” When faced with a person overdosing, the decision by others present of whether to call emergency services may be critical to the person’s survival. Several other participants registered suspicion of emergency responders, citing legal dangers, hostility and stigmatizing attitudes, which may inhibit those in a position to call emergency and medical services from doing so.

### Legal concerns

In Huntington, we heard rumors about a proposed ‘three-strike rule’ in a nearby Ohio town: emergency responders would only be dispatched on overdose calls if the victim had completed mandatory community service after an overdose reversal and would not respond for a third overdose. The proposal was not upheld [42] but fears about being denied access to care nevertheless remained. Worries about legal repercussions failed to deter some from seeking aid for overdoses. Christine reported that a heroin overdose in Huntington could result in a ticket but that she still wanted emergency response:

And if I don’t come back within 3 to 5 min after you hit me with that shot, you call 911. I don’t care. I’ll take… Because Huntington tickets you. They will write you a ticket. If you overdose on heroin and they have to show up to take care of you, they will write you a ticket and you have to go to court over it.

We found no evidence that Huntington was ticketing individuals for overdose responses, but this perception may nonetheless discourage some from calling emergency services. Others reported that calling emergency services for an overdose reversal would result in charges against the homeowner:

…Say it’s just me and Richard here and he overdoses – well, okay, this apartment is in his name but we all are like a family. But anyhow, and if I would overdose and die in here they’re going to charge him with manslaughter.

Q: Even though he didn’t supply the drug?

A: No, because it’s his house.

–Laura from Huntington, in her thirties, injecting for three months.

West Virginia is one of twenty US states with a drug-induced homicide law (so-called Len Bias laws) on the books, which can result in penalties of up to life imprisonment for manufacture or delivery of a controlled substance [43]. Under West Virginia’s law, sharing drugs with someone who dies of an accidental overdose can result in a murder conviction [44]. There were 91 overdose-homicide prosecutions in West Virginia between 2011 and 2016 and in 2017 the state passed a new law strengthening these provisions [45]. These laws effectively put the responsibility and blame for overdose deaths upon PWID and may deter those at the scene from calling emergency services despite the existence of West Virginia’s Good Samaritan law.

### Emergency responder attitudes

Kevin, from a small town in Kanawha County, reported being stigmatized while taking a friend to a fire station for emergency aid:

[…] I took her down [to the fire station], I got out of the car screaming, “She’s overdosing!” And they came over and the firefighters were fucking with me because I was pretty high.[…] like, “You’re not going to fall on us next are you?” She’s over there dying and you’re over here joking with me because my legs are wobbly? And you know they had to hit her one time [inject one dose of naloxone] I think, and she came back and they asked her if she wanted to go to the hospital and she looked at me and I was like, “You don’t really need to go.” [Them] “You want to take his word for it? He’s the one who let this happen to you,” blah, blah, blah. I mean it didn’t help anything at the time really.

Others reported strongly negative, stigmatizing experiences suffered while being treated for an overdose. Tina, from a small town in Kanawha County, has several conditions that led her to rely on extensive medical and extra-medical pain management and spoke about what she experienced while being treated for attempted suicide by overdose:

[…] there was a time, not that long ago actually, [that I overdosed]—it was attempted suicide and I used the heroin to do it.[…] Woke up, the paramedics were around me and I heard a cop—he didn’t know I could hear him—but he said, ‘You should have just let the junky bitch die.’

## Barriers to prevention services

In 2015, county government-sponsored NSPs were piloted in Kanawha and Cabell Counties, with physical locations at the Charleston and Huntington branches of the county health department [23]. Dr. Michael Brumage, former Executive Director of the Kanawha-Charleston Health Department, explained that support for a needle and syringe program within the city depended upon public endorsement by local police leaders:

As long as the police were vocally supporting the program there was little pushback with the people [in Charleston], and we always heard a little bit of background noise in terms of resistance and stigma that was still out there, but as long as the police were supporting us that was kept under wraps. And all of that changed when we had a new police chief […] there was a sudden and very dramatic shift in attitudes. (Late 2017 into early 2018) [46]

Meanwhile, in 2017 Dr. Brumage began experiencing opposition from outside Charleston:

[…] outside the city limits people were quite hostile. And you know Charleston is the largest town or city in Kanawha County but in surrounding municipalities there was a lot of resistance and people were not happy about our program in the surrounding communities.

This sensitive balance limited the services that the Charleston NSP was able to provide. Although it provided classes in reversing opioid overdose using naloxone, its donated supply of naloxone auto-dispensers was light and episodic. This curbed the NSP’s ability to reach those who needed them and potentially increased the burden on emergency services.

The Charleston NSP operated on a one-for-one-plus model of syringe access at the time of our visit in September 2017: new clients received an amount of syringes based on self-reported daily use, up to a maximum of 30. However, while government-issued identification was not required, losing or not having an NSP registration card resulted in a mandated five needle reduction in supplied syringes, while failing to return syringes bumped clients down to a lower limit of 10 syringes that could be worked up from over time by returning syringes over subsequent visits. The Charleston NSP operations manual admitted the political constraints on harm reduction services, stating “Although the needs based negotiated model is better at increasing syringe coverage, programs may have other reasons for using a one-for-one plus exchange model. In some communities, it is more politically palatable to assure everyone that the program is exchanging needles as opposed to distributing them.” [47]

Although we did not witness syringe sharing in West Virginia, supply access was limited, potentially encouraging sharing and likely heightened following the Charleston NSP’s closure, increasing the risk of both overdose and HIV/HCV transmission among the NSP’s clients [48]. This may be particularly true for individuals living outside of Charleston or Huntington. Most participants denied sharing syringes with others but recent outbreaks of HIV in the state have included injection drug use transmission [25, 49] and indicates that some sharing of injecting equipment is taking place. The evidence base for syringe exchange supports maximizing the number of syringes distributed so that all injections are performed with new syringes; low threshold, need-based distribution models allow for secondary exchange (in-person clients receiving and distributing syringes to their substance using peers), thus reaching populations who might not access the exchange directly [50–52].

Despite these drawbacks, participants were enthusiastic about the service and rarely expressed criticisms. All interviews were conducted outside of the facility itself, which may reduce the likelihood of an influenced response. Rebecca, in her 30s, using heroin for two to three years, said:

I love the exchange. I was the second person that ever went to it.[…] And I actually promote because you know if you’re gonna do [heroin] like the most unsafe way ever, you need to be safe about the way you do it. Like clean needles and, you know, you don’t share, and I’m all about promoting healthy – healthy shooting.

The Charleston NSP saw 15,521 total visits in 2017 by a total of 5,559 unique individuals [53], an average of nearly 300 per week—an immense number of visits for a relatively small health services program staffed mainly by volunteers. At the time of our visit, the NSP was operating only one day per week, which compounded the challenges of serving their clients. Despite these difficulties, clients were roomed individually, offered health services and treated with dignity and respect. The staff was sympathetic and had strong personal relationships with clients. The operations manual stated that education and counseling could include ‘safer injection practices and vein care’ but due to time and political constraints, as well as a lack of experience, NSP volunteers could not teach safer injection techniques as originally planned. Ultimately, local political changes resulted in additional restrictions being imposed upon the NSP. Some of these restrictions, such as requiring blood tests as a condition of access, the NSP considered unethical and contributed to the decision to permanently close the NSP in 2018 rather than compromise its values [48, 54].

## Knowledge transmission

The novel spread of injected heroin and fentanyl occurring in WV has created an unusual situation where, unlike urban areas with long established heroin use, there are few people who have been injecting heroin long term to advise novices on harm reduction techniques [55] nor large numbers of peers in recovery who have successfully moved out of dependent heroin use to provide insight and guidance [56]. We found low levels of knowledge about safe injection practices among participants expressed in interviews and directly observed in video analyses.

Our sample was largely unfamiliar with proper injection techniques and much injection equipment was under-sourced or unavailable even before the closure of the NSP. Improper filtration technique was common and may be particular to the region. Specifically, poor filtration practices may damage needles or allow insoluble particles into the bloodstream—during several filmed injection sequences needles pushed through instead of resting on filters, potentially damaging the needle and limiting filtration effectiveness.

Heroin solution was often prepared with a cotton already in the cooker rather than added just prior to being drawn into the syringe. This practice may have encouraged the widespread reuse of filters that we witnessed. Among many PWIH, ‘pounding cottons’ or doing a ‘cotton shot’ is resorted to when heroin is unavailable or when experiencing withdrawal symptoms, sometimes shared between users as part of a ‘moral economy’ [38], but it appeared to be an everyday practice in our sample. Respondents also used the same filter for multiple injections, which has been associated with the condition colloquially referred to as ‘cotton fever’ [57].

Self-syringe-reuse was reported, which may be associated with viral disease transmission through misplacing or mixing up syringes with others [58]. Christine described the difficulties of reusing syringes:

… [T]hey get dull and they get spurs on the end of them and that’s what causes the big red marks that you see on people a lot, and causes abscesses and stuff… […] And then sometimes like where it’s not sharp enough of a point it won’t puncture the vein, so you would literally have to take it and stab your vein and then you have to hear that pop to know that you’re inside your vein…

Several injections used extremely small amounts of water (10–15 units) because of beliefs that using more water reduced the overall dose strength. Due to cost constraints, sterile water was unavailable from the NSP which may have led to the substitution of other fluids, water sharing or using unsafe water sources [59]. Heating of heroin solution was not uniform, with many reporting that heroin did not require heat to go into solution. Cookers were frequently reused, either for a ‘rinse’ or for subsequent injections and we did not see cookers cleaned before or after use.

## Role Reversal

Close family relationships are considered a hallmark of Appalachian society [12], and the overdose crisis in West Virginia reflects this history while simultaneously introducing new dynamics around drug use within families. Many respondents indicated that they frequently used heroin or other drugs with family members, or had been introduced to drug use by family. Unusually, younger family members had introduced several older participants to heroin:

[…] I started five years ago. I have a lot of back pain. My daughter was using at the time, which I didn’t know. She came in to visit me one day and I was just – just really in a lot of pain that day. And she pulled that [heroin] out and she told me, ‘You know, Mom, if you do this, you know you’ll feel better.’ And I did and you know once that – I mean I felt so good after she gave me that shot […] Like I said, the pain’s gone. You don’t have to feel it or worry about it, it’s just awesome. I mean when you hurt as bad as what I hurt and doing a shot of heroin and—it’s gone. But only thing is now where I was controlling it, it’s controlling me more now than it used to.

–Julie

This inversion of the usual dynamic was seen among several older participants who had begun using heroin in the last several years. Christine believed introducing older family members to heroin was a natural response to seeing family members in pain:

[…] I think that’s what a lot of it is, is the kids nowadays see that their parents are in pain and they introduced their parents to it.[…] Hey, this will help you more than your pain medicine does. And parents get hooked on to it. And it’s just like one day sitting down and having a family dinner, and it’s like everybody sits down and has family shots or family lines…

Others relied on family members for income generation, drug provision or injection assistance, or in some cases, like Eric from Charleston, in his forties and injecting for three months, all three:

…You know because the daughter, the oldest daughter, both daughters work the streets or the truck stops. And they help their brother […] Because he can’t go work the streets like the girls can.[…] They’ll help the mother. Heck, they’ve been helping me. Plus, the grandma!

## Study limitations

Qualitative research is explorative and has some inherent limitations due to convenience sampling and unmeasured biases. The data provides a snapshot of views and experiences that can generate hypotheses. It is possible that conducting participant recruitment in a health care or public health setting may have influenced respondents to answer in ways deemed desirable by service providers. However, all of the interviews took place outside of these environments, in participants’ homes or other locations the participant felt safe, allowing cross-checking of responses.

## Discussion

An analysis of CDC cause of mortality data shows that both dependence on illicit drugs and deaths from drug overdose tend to be higher in US states where there is greater income inequality [60]. By median household income, West Virginia is one of the poorest states in the USA as a whole yet income inequalities are steep across the state [61]. Such inequalities create a risk environment conducive to premature death from many causes [33], including so-called diseases of despair: drug overdose, suicide and alcoholic liver disease or cirrhosis [62, 63]. Increases in mortality from many diseases were already claiming the lives of West Virginians from the early 1980s and the introduction of an easily accessible supply of opioids from the late 1990s onwards, first with prescription pills, then heroin and fentanyl, into this risk environment has provided another pathway to premature death.

Efforts to alleviate the opioid crisis that address only its symptoms rather than its underlying causes will give only temporary and localized relief. The social and economic structural factors that nurture problematic drug use need to be addressed through economic development [33, 63] and concerted efforts to bring about social change. Such economic and social development needs to take place with the good of West Virginia’s own citizens in mind, not simply in the interests of out-of-state landowners and employers.

Industrial injuries are common in the state’s extractive and chemical industries and are often blamed for the high demand for prescription opioid pills for pain relief in West Virginia [64]. However, workplace accidents and injuries are not inevitable; their frequency and severity are influenced by many factors, including the type of safety regulations and inspections implemented [65, 66]. Prevention of occupational injuries could also help reduce the demand for opioids. Checking the power of corporations in local politics by emphasizing healthy, safety and environmental concerns and requiring companies to be responsive to local needs is just a first step. Employee-owned and accountable enterprises should be encouraged and given priority over out-of-state corporate interests. Evidence shows that greater participation in workplace decision-making and ownership are linked to improved outcomes in both employee health and productivity [67, 68].

The provision of treatment services accessible to the state’s largely rural population is vital and access to opioid treatment services may have improved since this research was carried out. In January 2018, approval was given for methadone treatment to be provided through Medicaid, but a longstanding moratorium on new methadone clinics in the state has blunted the impact of this approval. A small positive sign is the fall in opioid overdoses in the state from a peak of 874 in 2017 to a provisional count of under 700 in 2019 although there may be underreporting [69]. Many of these fatal events are preventable, but the willingness of prosecutors in the state to bring drug-induced homicide charges may have a significant impact on PWIH seeking aid in cases of overdose. Policymakers urgently need to address the conflict between having both a medical Good Samaritan law and a drug-induced homicide law on the books in order to reduce the state’s high rate of drug overdose deaths.

Measures of compassion and generosity show West Virginia to be rich in these qualities despite relative poverty yet PWIH in southern West Virginia described an often unsupportive and at times hostile risk environment. Hostility toward PWIH and harm reduction workers may increase the risk of overdose. Negative experiences, including from some emergency responders, and fears of punitive legal consequences from calling these services may deter users from seeking the help they need to survive overdoses or exit from drug use. Participants described highly stigmatizing responses to their calls for help and a lack of compassion from lawmakers, some of whom saw death from overdose as a desirable outcome. A new model for managing health emergencies without the presence of law enforcement is desperately needed, evidenced by several high-profile police killings of individuals dealing with personal mental health crises across the US in 2020 [70, 71].

Recent work on the opioid epidemic in a white affluent East Coast community has shown contrasting media representations of opioid dependence and overdose according to race, where white use has been framed in terms of victimization and blamelessness in the face of a biological disease of addiction. This contrasted with previous criminalizing and condemnatory narratives of urban Latino and Black opioid use [72]. However, in West Virginia, the response to opioid overdose was based more on class than race and poor white people were commonly blamed for their opioid dependence and overdoses. This resembled a pattern of criminalizing poor whites using crystal methamphetamine found in other ethnographic work in Appalachia [72, 73].

Antipathy did not stop at PWIH themselves but was also aimed at those trying to help them through the provision of harm reduction services. Awareness of potential opposition initially limited the activities of Charleston’s primary needle and syringe program, such as from providing larger numbers of syringes, but this turned to hostility in the campaign that ultimately culminated in its closure in 2018 after the research visit. The story of the NSP’s closure has been told elsewhere [48] but relevant to understanding the local risk environment are the power inequalities that underlay it. Charleston’s mayor in 2017, Danny Jones, used his daily radio show to denounce the program, adding to public hostility toward users and harm reduction workers. The NSP building was opposite and considered a threat to the newly renovated civic center, a $100 million investment and it seems that the interests of PWIH and public health were sacrificed to protect these interests [54].

The closure of Charleston’s NSP is likely to have important implications for the health of people who inject drugs in Charleston. The Charleston NSP fostered a climate of respect between city social services and the marginalized community of people who use drugs. No other equivalent service without constraints on syringe access and supply currently exists in the city to help PWIH. Overdose prevention sites have demonstrated success in several international contexts at preventing overdose among the individuals who access these services [74]. These sites also provide a unique opportunity to extend other health and social services to all people who use drugs, improve health outcomes and rates of drug treatment entry, provide job opportunities for peers in recovery and create a trusted touchpoint between people who use drugs and their communities [75–78]. However, as aggressive federal actions against the establishment of an overdose prevention site in Philadelphia show, there is significant, entrenched opposition to these facilities among many segments of law enforcement, and political hostility in West Virginia would likely be even greater than for an NSP.

Compassion fatigue, which can result from providing empathetic care and seeing clients continue to suffer [79], and burnout, from prolonged work stress, may be significant factors impacting emergency responders treating overdose cases [80]. Jan Rader, Huntington’s Fire Chief, reported at a February 2017 meeting of the West Virginia Association of Health Departments that first responders were seeing 5–6 overdose calls per 12-h shift and she was particularly concerned about burnout among her staff. As well as negative impact on health care workers themselves, both compassion fatigue and burnout can impair empathy for clients and the quality of care given [80]. John C Linton, Associate Vice President and Dean of the West Virginia University School of Medicine-Charleston and an emergency medical technician, helped to develop an intervention to reduce critical incident stress among pre-hospital emergency workers in West Virginia in the 1990s [81]. As a close observer of emergency service personnel during WV’s opioid epidemic, Professor Linton has witnessed initial compassion toward PWIH sometimes turning to irritability and impatience when they repeatedly require overdose reversal treatment and may not always appreciate the intervention [82].

In the context of an impoverished state’s scarce resources, this high demand for emergency services results in funding shortages for other urgent needs, creating resentment and blame. The perception of some providers that dependent drug use is a choice rather than a relapsing medical condition underpins a belief that anger toward PWIH is an appropriate response.

The state proposed a public education campaign to expand awareness of substance use disorder as a treatable disease and address misinformation and associated stigma [83]. The stigma experienced by PWIH in West Virginia, led and reinforced by some of those involved in policymaking, the criminal justice system and emergency services, will hamper rather than help the efficacy of the state’s response to the overdose crisis. These public education campaigns, framed within the local culture of mutual aid, support and compassion, could appeal to the public to help PWIH, to enable PWIH to help each other and to discourage measures that deter mutual assistance. Greater provision of overdose prevention education and resources such as naloxone for peer distribution and use could be promoted as a way of helping PWIH to reverse overdoses and alleviate the burden on emergency services. Outside of drug-related and public education interventions, increased monetary support and expanded healthcare access for low income families and children can help address some of the wealth and health disparities experienced by this population.

While essential for reducing mortality, measures that address the effects of drug use alone are not enough for safeguarding longer term public health. Although opioid overdoses have fallen in West Virginia between 2017 and 2019, since 2015 there has been a steady growth of deaths involving psychostimulants (mainly methamphetamine) [69], suggesting that the drivers of demand are not drug specific and cannot be blamed on vicissitudes in supply alone. This new wave of the overdose crisis underlines the urgency of addressing the deeper causes that feed high-risk patterns of drug use beyond the drugs and drug use itself.

## Data Availability

Data and materials are of a sensitive nature and are consequently not available to those outside the study team. A US Federal Certificate of Confidentiality issued by the National Institutes of Health/National Institute on Drug Abuse protects the data and its collection.

## References

[1] Scholl L, Seth P, Kariisa M, Wilson N, Baldwin G (2019). Drug and opioid-involved overdose deaths-United States, 2013–2017. MMWR Morb Mortal Wkly Rep.

[2] Seth P, Scholl L, Rudd RA, Bacon S (2018). Overdose deaths involving opioids, cocaine, and psychostimulants-United States, 2015–2016. MMWR Morb Mortal Wkly Rep.

[3] West Virginia Department of Health and Human Resources. West Virginia drug overdose deaths historical overview 2001–2015 [Internet]. Charleston, WV; 2017 Aug [cited 2018 Aug 21] p. 25. https://dhhr.wv.gov/oeps/disease/ob/documents/opioid/wv-drug-overdoses-2001_2015.pdf

[4] National Center for Health Statistics (2018). Centers for Disease Control and Prevention.

[5] U.S. Census Bureau QuickFacts: UNITED STATES [Internet]. [cited 2018 Aug 22]. https://www.census.gov/quickfacts/fact/table/wv,cabellcountywestvirginia,kanawhacountywestvirginia,huntingtoncitywestvirginia,charlestoncitywestvirginia,US/PST045217

[6] Benson C, Bishaw A. Poverty: 2017 and 2018. U.S. Department of Commerce, U.S. Census Bureau; 2019 Nov. (American Community Survey Briefs). Report No.: ACSBR/18-02(RV).

[7] Beaver PD, Keefe SE (1988). Appalachian cultural systems, past and present. Appalachian mental health.

[8] Bell SE, York R (2010). Community economic identity: the coal industry and ideology construction in West Virginia. Rural Sociol.

[9] Spence B, Kunkel C, Schewel E, Boettner T, Martin L. Who owns West Virginia? West Virginia Center on Budget & Policy. 2013;13.

[10] Parker L. A century of controversy, accidents in West Virginia’s chemical valley in lead-up to spill. National Geographic. 2014;

[11] Fox J (1999). Mountaintop removal in West Virginia: an environmental sacrifice zone. Organ Environ.

[12] Coyne CA, Demian-Popescu C, Friend D (2006). Social and cultural factors influencing health in Southern West Virginia: a qualitative study. Prev Chronic Dis.

[13] West Virginia Civic Health Index [Internet]. National Conference on Citizenship. [cited 2019 Aug 12]. https://ncoc.org/research-type/west-virginia-civic-health-index/

[14] 50 Most Generous Cities in America [Internet]. Barna Group. 2016 [cited 2019 Aug 12]. https://www.barna.com/research/50-most-generous-cities-in-america/

[15] Mars SG, Bourgois P, Karandinos G, Montero F, Ciccarone D (2014). “Every ‘never’ I ever said came true”: transitions from opioid pills to heroin injecting. Int J Drug Policy.

[16] Centers for Disease Control and Prevention. U.S. County Prescribing Rates, 2016 [Internet]. U.S. County Prescribing Rates, 2016 | Drug Overdose | CDC Injury Center. 2017 [cited 2018 Aug 22]. https://www.cdc.gov/drugoverdose/maps/rxcounty2016.html

[17] Young AM, Havens JR (2012). Transition from first illicit drug use to first injection drug use among rural Appalachian drug users: a cross-sectional comparison and retrospective survival analysis. Addiction.

[18] Havens JR, Oser CB, Knudsen HK, Lofwall M, Stoops WW, Walsh SL (2011). Individual and network factors associated with non-fatal overdose among rural Appalachian drug users. Drug Alcohol Depend.

[19] Havens JR, Lofwall MR, Frost SD, Oser CB, Leukefeld CG, Crosby RA (2013). Individual and network factors associated with prevalent hepatitis C infection among rural Appalachian injection drug users. Am J Public Health.

[20] Keyes KM, Cerdá M, Brady JE, Havens JR, Galea S (2014). Understanding the rural-urban differences in nonmedical prescription opioid use and abuse in the United States. Am J Public Health.

[21] Babcock C, Rockich-Winston N, Booth C (2017). Bringing naloxone to ground zero: Huntington, West Virginia. J Am Pharm Assoc.

[22] Schneider KE, O’Rourke A, White RH, Park JN, Musci RJ, Kilkenny ME (2020). Polysubstance use in rural West Virginia: Associations between latent classes of drug use, overdose, and take-home naloxone. Int J Drug Policy.

[23] Bixler D, Corby-Lee G, Proescholdbell S, Ramirez T, Kilkenny ME, LaRocco M (2018). Access to syringe services programs-Kentucky, North Carolina, and West Virginia, 2013–2017. MMWR Morb Mortal Wkly Rep.

[24] W.-O.W.V.H.D. HIV Cluster Identified in Ohio County [Internet]. 2018 [cited 2019 Mar 14]. https://www.ohiocountyhealth.com/news/hiv-cluster-identified-in-ohio-county/

[25] West Virginia Department of Health and Human Resources Bureau for Public Health. Outbreak of human immunodeficiency virus (HIV) linked to injection drug use. Human immunodeficiency virus (HIV) and acquired immune deficiency syndrome (AIDS). 2019.

[26] Office of Epidemiology & Prevention Services. Outbreak of Human Immunodeficiency Virus (HIV) Linked to Injection Drug Use [Internet]. 2020 [cited 2020 Apr 17]. https://oeps.wv.gov/hiv-aids/pages/default.aspx

[27] Rhodes T (2009). Risk environments and drug harms: a social science for harm reduction approach. Int J Drug Policy.

[28] Mars SG, Ondocsin J, Ciccarone D. Toots, tastes and tester shots: user accounts of drug sampling methods for gauging heroin potency. Harm Reduction Journal [Internet]. 2018 Dec [cited 2018 Jul 6];15(1). https://harmreductionjournal.biomedcentral.com/articles/10.1186/s12954-018-0232-z 10.1186/s12954-018-0232-z PMC5956544 29769132

[29] Ciccarone D, Ondocsin J, Mars SG (2017). Heroin uncertainties: exploring users’ perceptions of fentanyl-adulterated and-substituted ‘heroin’. Int J Drug Policy.

[30] Mars SG, Ondocsin J, Ciccarone D (2017). Sold as Heroin: Perceptions and Use of an Evolving Drug in Baltimore, MD. J Psychoactive Drugs.

[31] Ciccarone D (2017). Fentanyl in the US heroin supply: a rapidly changing risk environment. Int J Drug Policy.

[32] McKnight C, Des Jarlais DC (2018). Being “hooked up” during a sharp increase in the availability of illicitly manufactured fentanyl: Adaptations of drug using practices among people who use drugs (PWUD) in New York City. Int J Drug Policy.

[33] Dasgupta N, Beletsky L, Ciccarone D (2018). Opioid crisis: no easy fix to its social and economic determinants. Am J Public Health.

[34] Meit M, Heffernan M, Tanenbaum E, Hoffmann T. Appalachian diseases of despair [Internet]. The Walsh Center for Rural Health Analysis; 2017 Aug [cited 2018 Aug 31]. https://www.arc.gov/assets/research_reports/AppalachianDiseasesofDespairAugust2017.pdf

[35] Rhodes T (2002). The ‘risk environment’: a framework for understanding and reducing drug-related harm. Int J Drug Policy.

[36] Ciccarone D, Bourgois P (2003). Explaining the geographical variation of HIV among injection drug users in the United States. Subst Use Misuse.

[37] Van Handel MM, Rose CE, Hallisey EJ, Kolling JL, Zibbell JE, Lewis B (2016). County-level vulnerability assessment for rapid dissemination of HIV or HCV infections among persons who inject drugs, United States. JAIDS.

[38] Bourgois P, Schonberg J (2009). Righteous dopefiend.

[39] Fessel JN, Mars S, Bourgois P, Ciccarone D. Into the epistemic void: using rapid assessment to investigate the opioid crisis. In: Shukla MB a. RK, editor. Inside ethnography: researchers reflect on the challenges of reaching hidden populations. Berkeley: University of California Press; Forthcoming.

[40] Castillo T, Ciccarone D, Davidson PJ, Davis C, Beletsky L, LaRocco M, et al. Reviews of the 2018 WVDHHR-BPH Report of the Kanawha-Charleston Health Department Harm Reduction Syringe Services Program. Distributed to the Kanawha-Charleston Board of Health; 2018.

[41] Cicero TJ, Ellis MS, Surratt HL, Kurtz SP (2014). The changing face of heroin use in the United States: a retrospective analysis of the past 50 years. JAMA Psychiatry.

[42] Wootson Jr. CR. One politician’s solution to the overdose problem: Let addicts die. Washington Post [Internet]. 2017 Jun 30 [cited 2019 Aug 13]. https://www.washingtonpost.com/news/to-your-health/wp/2017/06/28/a-council-members-solution-to-his-ohio-towns-overdose-problem-let-addicts-die/

[43] Beletsky L. America’s Favorite Antidote: Drug-Induced Homicide in the Age of the Overdose Crisis. SSRN Journal [Internet]. 2018 [cited 2019 Aug 12]. https://www.ssrn.com/abstract=3185180

[44] Goldensohn R (2018). They shared drugs.

[45] LaSalle L. An overdose death is not murder: why drug-induced homicide laws are counterproductive and inhumane. The Drug Policy Alliance. 2017;

[46] Interview with Dr. Michael Brumage. 2018.

[47] Kanawha-Charleston Health Department (2017). Harm reduction syringe services program: program procedure manual.

[48] Allen ST, Grieb SM, O’Rourke A, Yoder R, Planchet E, White RH (2019). Understanding the public health consequences of suspending a rural syringe services program: a qualitative study of the experiences of people who inject drugs. Harm Reduct J.

[49] Evans ME, Labuda SM, Hogan V, Agnew-Brune C, Armstrong J, Karuppiah ABP (2018). Notes from the field: HIV infection investigation in a rural Area-West Virginia, 2017. MMWR Morb Mortal Wkly Rep.

[50] Kral AH, Anderson R, Flynn NM, Bluthenthal RN (2004). Injection risk behaviors among clients of syringe exchange programs with different syringe dispensation policies. JAIDS.

[51] Wodak A, Cooney A (2006). Do needle syringe programs reduce HIV infection among injecting drug users? A comprehensive review of the international evidence’, substance use and misuse. Subst Use Misuse.

[52] Voytek C, Sherman SG, Junge B (2003). A matter of convenience: factors influencing secondary syringe exchange in Baltimore, Maryland, USA. Int J Drug Policy.

[53] Crouch BJ, Gupta R, Haddy L. 2018 Evaluation Report of the Kanawha-Charleston Health Department Harm Reduction Syringe Services Program [Internet]. 2018 p. 62. https://dhhr.wv.gov/bph/Documents/HarmReductionReports/2018%2520Evaluation%2520Report%2520KCHD%2520HRSSP%2520May%25202018%252005.11.18.pdf

[54] Katz J (2018). Why a city at the center of the opioid crisis gave up a tool to fight it.

[55] Murphy S, Waldorf D (1991). Kickin’down to the street doc: shooting galleries in the San Francisco Bay Area. Contemp Drug Probs.

[56] Satinsky EN, Doran K, Felton JW, Kleinman M, Dean D, Magidson JF (2020). Adapting a peer recovery coach-delivered behavioral activation intervention for problematic substance use in a medically underserved community in Baltimore City. PLoS ONE.

[57] Torka P, Gill S (2013). Cotton fever: an evanescent process mimicking sepsis in an intravenous drug abuser. J Emerg Med.

[58] Ciccarone D, Bourgois P (2016). Injecting drugs in tight spaces: HIV, cocaine and collinearity in the Downtown Eastside, Vancouver, Canada. Int J Drug Policy.

[59] Harris M, Scott J, Hope V, Wright T, McGowan C, Ciccarone D (2020). Navigating environmental constraints to injection preparation: the use of saliva and other alternatives to sterile water among unstably housed PWID in London. Harm Reduct J.

[60] Wilkinson RG, Pickett K (2011). The spirit level: why greater equality makes societies stronger.

[61] US Bureau of the Census. Current Population Survey. Annual Social and Economic Supplements [Internet]. 2019. https://www2.census.gov/programs-surveys/cps/techdocs/cpsmar19.pdf

[62] Case A, Deaton A (2017). Mortality and morbidity in the 21st century. Brookings papers on economic activity.

[63] Meit M, Heffernan M, Tanenbaum E, Hoffmann T. Appalachian diseases of despair. Appalachian Regional Commission; 2017.

[64] Drug Enforcement Administration. DEA 360 Strategy Reach and Impact Report: Charleston. Department of Justice; 2018.

[65] Poplin GS, Miller HB, Ranger-Moore J, Bofinger CM, Kurzius-Spencer M, Harris RB (2008). International evaluation of injury rates in coal mining: a comparison of risk and compliance-based regulatory approaches. Saf Sci.

[66] Levine DI, Toffel MW, Johnson MS (2012). Randomized government safety inspections reduce worker injuries with no detectable job loss. Science.

[67] Bosma H, Marmot MG, Hemingway H, Nicholson AC, Brunner E, Stansfeld SA (1997). Low job control and risk of coronary heart disease in Whitehall II (prospective cohort) study. BMJ.

[68] Conyon MJ, Richard B. Shared Modes of Compensation and Firm Performance: UK Evidence. NBER Working Paper. 8448.

[69] Centers for Disease Control and Prevention: National Center for Health Statistics. 12 Month-ending Provisional Number of Drug Overdose Deaths by Drug or Drug Class [Internet]. 2020 [cited 2020 Jan 30]. https://www.cdc.gov/nchs/nvss/vsrr/drug-overdose-data.htm

[70] What to Know About Daniel Prude’s Death. The New York Times [Internet]. 2020 Sep 4 [cited 2020 Sep 11]. https://www.nytimes.com/2020/09/04/nyregion/rochester-daniel-prude.html

[71] Associated Press in Lafayette Louisiana. Louisiana shooting: police killing of Black man sparks outrage and protests. the Guardian [Internet]. 2020 Aug 23 [cited 2020 Sep 11]. https://www.theguardian.com/us-news/2020/aug/23/louisiana-shooting-police-kill-black-man-trayford-pellerin

[72] Mendoza S, Rivera AS, Hansen HB (2019). Re-racialization of addiction and the redistribution of blame in the white opioid epidemic. Med Anthropol Q.

[73] Garriott W (2013). You can always tell who’s using meth": Methamphetamine addiction and the semiotics of criminal difference. Addict Traj.

[74] Potier C, Laprévote V, Dubois-Arber F, Cottencin O, Rolland B (2014). Supervised injection services: what has been demonstrated? A systematic literature review. Drug Alcohol Depend.

[75] Wood E, Tyndall MW, Zhang R, Montaner JS, Kerr T (2007). Rate of detoxification service use and its impact among a cohort of supervised injecting facility users. Addiction.

[76] Wood E, Kerr T, Small W, Li K, Marsh DC, Montaner JS (2004). Changes in public order after the opening of a medically supervised safer injecting facility for illicit injection drug users. CMAJ.

[77] Pinkerton SD (2011). How many HIV infections are prevented by Vancouver Canada’s supervised injection facility?. Int J Drug Policy.

[78] Kennedy MC, Boyd J, Mayer S, Collins A, Kerr T, McNeil R (2019). Peer worker involvement in low-threshold supervised consumption facilities in the context of an overdose epidemic in Vancouver. Can Soc Sci Med.

[79] McHolm F (2006). Rx for compassion fatigue. J Christ Nurs.

[80] Medland J, Howard-Ruben J, Whitaker E. Fostering psychosocial wellness in oncology nurses: addressing burnout and social support in the workplace. In: Oncology nursing forum. 2004. 10.1188/04.ONF.47-54 14722587

[81] Linton JC, Kommor MJ, Webb CH (1993). Helping the helpers: the development of a critical incident stress management team through university/community cooperation. Ann Emerg Med.

[82] Interview with Dr. John C. Linton. 2020.

[83] Gupta R (2018). Opioid response plan for the state of West Virginia.

