# Correction to: Hostility, compassion and role reversal in West Virginia’s long opioid overdose emergency

**DOI:** 10.1186/s12954-021-00542-z

**Published:** 2021-08-30

**Authors:** Jeff Ondocsin, Sarah G. Mars, Mary Howe, Daniel Ciccarone

**Affiliations:** 1 Heroin in Transition Study, Department of Family and Community Medicine, UCSF, University of California, 500 Parnassus Avenue, Milberry Union East, 3rd Floor, San Francisco, CA 94143 USA; 2 Homeless Youth Alliance, PO Box 170427, San Francisco, CA 94117 USA

## Correction to: Harm Reduct J (2020) 17:74 10.1186/s12954-020-00416-w

After publication of the original article [1], the authors identified an error in Acknowledgements section. The below disclosures is missing.

"Dr. Ciccarone has received compensation in the past 36 months for: being on the Scientific Advisory Board for Celero Systems; providing expert testimony for Motley Rice LLP; being a one-time consultant for Arbor Pharmaceuticals; all outside the submitted work".

The original article has been corrected.
